# Stepwise Biogenesis of Subpopulations of Lipid Droplets in Nitrogen Starved *Phaeodactylum tricornutum* Cells

**DOI:** 10.3389/fpls.2020.00048

**Published:** 2020-02-11

**Authors:** Antoine Jaussaud, Josselin Lupette, Juliette Salvaing, Juliette Jouhet, Olivier Bastien, Marina Gromova, Eric Maréchal

**Affiliations:** ^1^ Laboratoire de Physiologie Cellulaire et Végétale, Université Grenoble Alpes, Centre National de la Recherche Scientifique, Commissariat à l'énergie atomique et aux énergies alternatives, Institut National de Recherche pour l’Agriculture, l’Alimentation et l’Environnement, IRIG, CEA-Grenoble, Grenoble, France; ^2^ Plant Research Laboratory, Department of Energy, Michigan State University, MI, East Lansing, USA; ^3^ Laboratoire Modélisation et Exploration des Matériaux, Université Grenoble Alpes, Commissariat à l'énergie atomique et aux énergies alternatives, IRIG, CEA-Grenoble, Grenoble, France

**Keywords:** diatoms, *Phaeodactylum*, Triacylglycerol, lipid droplet, lipids

## Abstract

Diatoms are unicellular heterokonts, living in oceans and freshwaters, exposed to frequent environmental variations. They have a sophisticated membrane compartmentalization and are bounded by a siliceous cell-wall. Formation of lipid droplets (LDs), filled with triacylglycerol (TAG), is a common response to stress. The proteome of mature-LDs from *Phaeodactylum tricornutum* highlighted the lack of proteins involved in early-LD formation, TAG biosynthesis or LD-to-LD connections. These features suggest that cytosolic LDs might reach a size limit. We analyzed the dynamics of LD formation in *P. tricornutum* (Pt1 8.6; CCAP 1055/1) during 7 days of nitrogen starvation, by monitoring TAG by mass spectrometry-based lipidomics, and LD radius using epifluorescence microscopy and pulse field gradient nuclear magnetic resonance. We confirmed that mature LDs reach a maximal size. Based on pulse field gradient nuclear magnetic resonance, we did not detect any LD-LD fusion. Three LD subpopulations were produced, each with a different maximal size, larger-sized LDs (radius 0.675 ± 0.125 µm) being generated first. Mathematical modeling showed how smaller LDs are produced once larger LDs have reached their maximum radius. In a mutant line having larger cells, the maximal size of the first LD subpopulation was higher (0.941 ± 0.169 µm), while the principle of stepwise formation of distinct LD populations was maintained. Results suggest that LD size is determined by available cytosolic space and sensing of an optimal size reached in the previous LD subpopulation. Future perspectives include the unraveling of LD-size control mechanisms upon nitrogen shortage. This study also provides novel prospects for the optimization of oleaginous microalgae for biotechnological applications.

## Introduction

Diatoms constitute a major group of unicellular photosynthetic heterokonts (or stramenopiles), living in oceans and freshwaters, predicted to be responsible for up to 20–25% global primary productivity, a contribution equivalent to that of all terrestrial rain forests (Field et al., 1998; Mann, 1999). Diatom anatomy has unique features including the presence of a rigid cell wall made of silica, called a frustule (De Tommasi et al., 2017). Centric diatoms are radially symmetric, such as the model species *Thalassiosira pseudonana* (Armbrust et al., 2004), whereas pennate diatoms are mainly based on a bilateral symmetry, such as the model species *Phaeodactylum tricornutum* (Bowler et al., 2008). Since *P. tricornutum* can grow without added silicate (Lewin, 1958) the fusiform or triradiate cell shape relies on the stiffness of a cell wall made of polysaccharides, predominantly a linear poly-α-(1→3) mannan decorated with sulfate ester groups and β-D-glucuronic residues, ultimately strengthened by the presence of silica (Le Costaouec et al., 2017).

**Figure 1:**
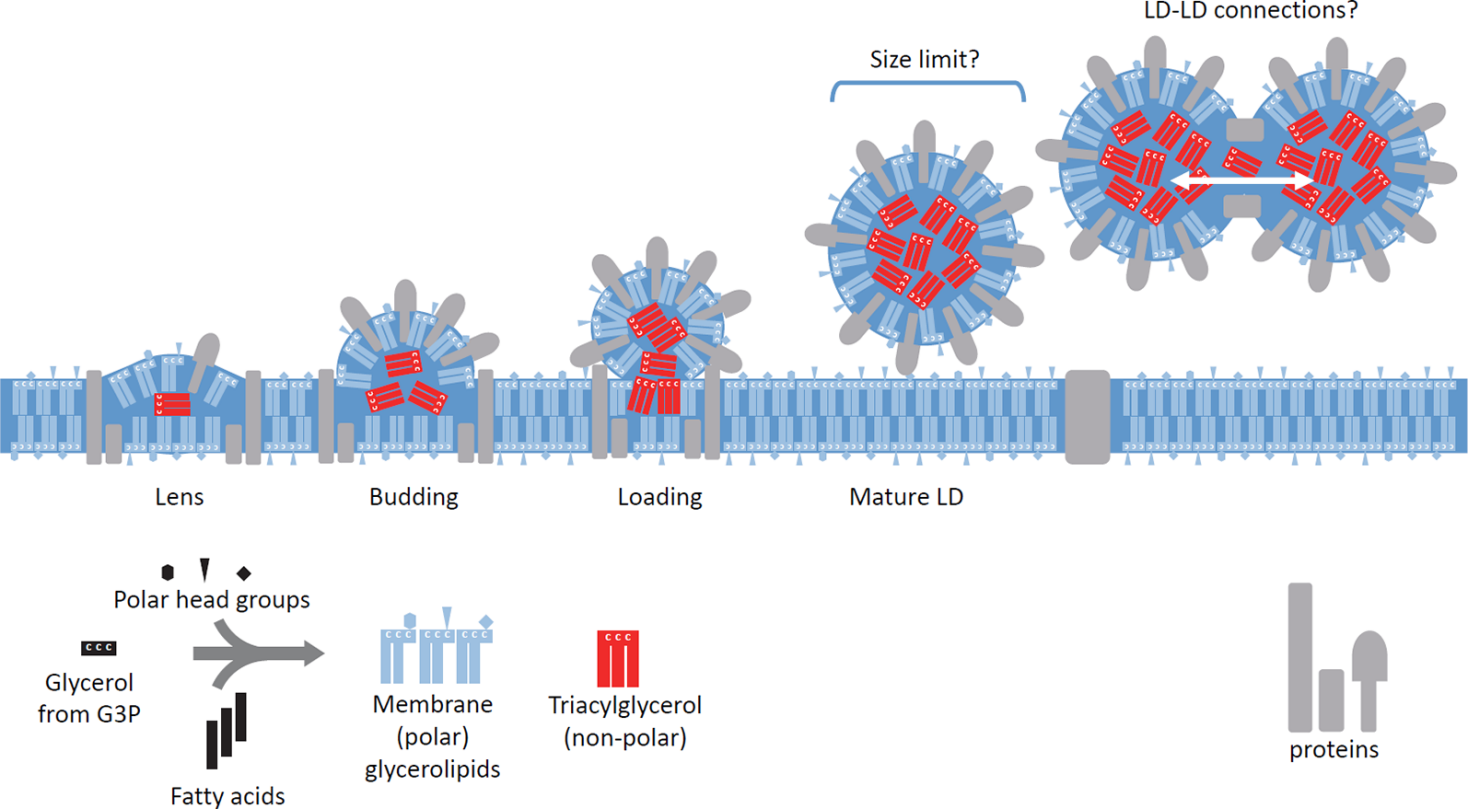
Stepwise biogenesis of lipid droplets in *Phaedoactylum*. The precise membrane platform producing cytosolic lipid droplets (LDs) in *Phaeodactylum* is still unknown, but proteomic and lipidomic analyses suggest a location at the outermost membrane of the plastid (epiplastidial membrane or EpM) or at the vicinity of the EpM. LDs are sub-spherical organelles loaded with triacylglycerol (TAG, non-polar glycerolipid), shown in red. TAG does not form lipid bilayers like other polar glycerolipids, shown in blue. The initial step (Lens) involves multiple proteins, including, on the one hand, enzymes producing TAG *using de novo* synthesized diacylglycerol or recycling available polar glycerolipids, and on the other hand, multiple structural proteins making a boundary to this LD-forming platform, like SEIPIN. Membrane thickening leads to the formation of a LD primordium (Budding), and the activity of TAG-synthesizing enzymes leads to the formation of a sub-spherical structure (Loading). The absence of homologues of proteins usually involved in LD biogenesis in late stages (Mature LDs) suggests that the LD is not connected anymore to its biogenetic platform. It is not known whether a size limit determines the production of mature LDs. The existence of connections between LDs by proteins that would act like CIDE proteins described in other eukaryotes, ensuring a mobility of TAG molecules from one LD to another, is also unknown.

Like most photosynthetic eukaryotes living in an environment subjected to frequent variations, diatoms have to cope with abiotic stresses of very diverse natures. An intense remodeling of glycerolipids leading to the formation of lipid droplets (LDs) is a common feature of the response of phytoplankton to stresses such as nutrients' starvation (Nguyen et al., 2011; Abida et al., 2015; Popko et al., 2016), high temperature (Yao et al., 2012; Alboresi et al., 2016), high light (Alboresi et al., 2016), exposure to nitric oxide (Dolch et al., 2017), hydrogen peroxide (Burch and Franz, 2016; Collins et al., 2016; Conte et al., 2018) or to a variety of chemicals (Kim et al., 2017; Wase et al., 2017; Conte et al., 2018; Wase et al., 2018). Glycerolipids consist of a three-carbon glycerol backbone (numbered *sn*-1, 2, and 3) esterified to fatty acids (FAs) at positions *sn*-1 and *sn*-2, which *sn*-3 position can be linked to a polar head. They make the bulk of cell membranes and each subcellular compartment contains a very precise glycerolipid composition. The nature of the polar head defines glycerolipid classes, such as the phospholipid phosphatidylcholine (PC) or the betain lipid diacylglyceryl hydroxymethyltrimethyl-β-alanine (DGTA), usually synthesized in the endoplasmic reticulum (ER) or the glycolipids monogalactosyldiacylglycerol (MGDG), digalactosyldiacylglycerol (DGDG) or sulfoquinovosyldiacylglycerol (SQDG) synthesized in plastids (Boudiere et al., 2012; Boudiere et al., 2014; Petroutsos et al., 2014; Li-Beisson et al., 2019). Stress-induced remodeling corresponds therefore to changes in the proportions of different glycerolipid classes, of the FAs contained in each class and sometimes modification of the subcellular location of these lipids within the cell. The most spectacular change is the production of triacylglycerol (TAG), by addition of a third FA at the *sn*-3 position of glycerol instead of a polar head. TAG destabilizes membranes and accumulates inside LDs (**Figure 1**), together with other neutral lipophilic components that can include sterol derivatives or carotenoids (Lupette et al., 2019).

**Figure 2:**
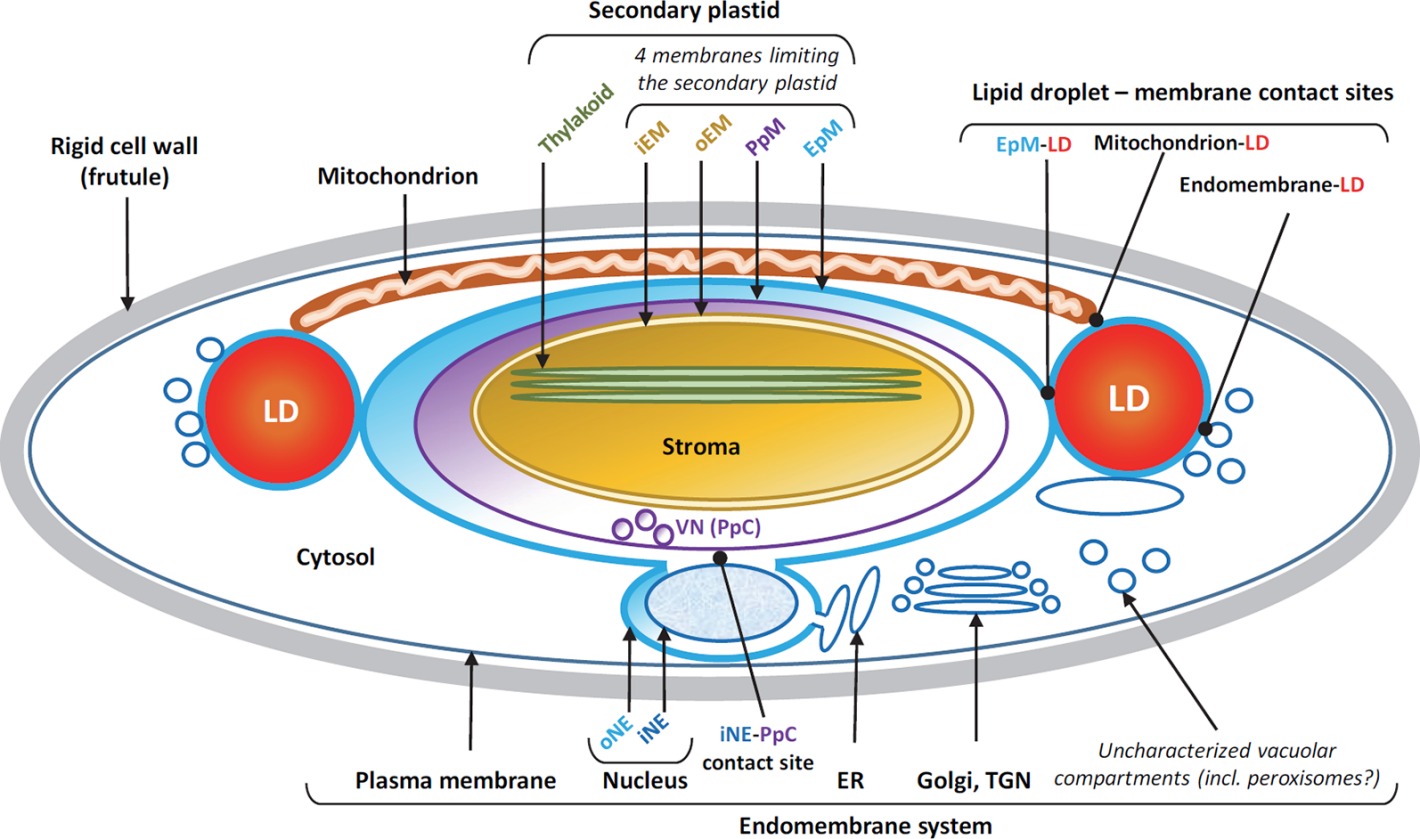
Membrane compartmentalization and lipid droplet (LD)-membrane contacts in the pennate diatom *Phaeodactylum*. Diatoms contain a secondary plastid deriving from a secondary endosymbiosis. Four membranes limit this plastid, from inside to outside: the inner (iEM) and outer envelope membranes (oEM), the periplastidial membrane (PpM), and the epiplastidial membrane (EpM). The PpM generates a vesicular network (VN) in the periplastidial compartment (PpC), with unknown function. The EpM is continuous with the outer nuclear envelope (oNE). At the level of the isthmus between the secondary plastid and the nucleus, a direct contact occurs between the PpM and the inner nuclear envelope (iNE). LDs, shown in red, are located in the cytosol on each side of the elongated plastid. Direct contact sites between LDs and membranes are observed with the EpM, the mitochondrion and uncharacterized endomembranes. It is likely that the EpM-LD contact site corresponds to the LD-forming platform, whereas LD-mitochondrion and LD-endomembrane contact sites correspond to catabolic platforms where fatty acids hydrolyzed from triacylglycerol might be exported for downstream degradation by beta-oxidation, or recycling for other purposes. The whole cell is bounded by a rigid cell wall, or frustule.

Recent lipidomic and proteomic study of the purified LD of nitrogen-starved *P. tricornutum* has allowed reconstructing basic features of the architecture of this organelle and has pointed some membrane compartments that could be involved in its biogenesis and function (Lupette et al., 2019). Since TAG derives from membrane glycerolipids, LD biogenesis requires a membrane for its initial formation by budding. In yeast or mammals, LD buds from the outer leaflet of the ER (Pol et al., 2014; Wilfling et al., 2014; Walther et al., 2017), whereas in photosynthetic algae such as *Chlamydomonas*, LD biogenesis involves the chloroplast envelope (Nguyen et al., 2011). Diatoms have a much more sophisticated intracellular membrane architecture, with a chloroplast acquired following a secondary endosymbiosis, known as a “secondary” or “complex plastid” (Janouskovec et al., 2010; Flori et al., 2016; Fussy and Obornik, 2018) (**Figure 2**). This secondary plastid is delineated by four membranes, the outermost one, or epiplastid membrane (EpM) being continuous with the nuclear envelope and therefore connected to the endomembrane system, including the ER (Flori et al., 2016; Cavalier-Smith, 2018) (**Figure 2**). Through this connection, the ER could possibly contribute to the lipid biogenesis of the EpM, by supplying some lipids necessary to build up this membrane (Petroutsos et al., 2014; Abida et al., 2015). The second outermost membrane, or periplastidial membrane has no apparent continuity with other cell systems and no hypothesis is currently proposed regarding its lipid biogenesis. A vesicular network was shown to protrude from the periplastidial membrane and a membrane contact site with the nuclear inner envelope membrane has been observed (**Figure 2**) (Flori et al., 2016; Cavalier-Smith, 2018); the function of these structures is still unknown. The two innermost membranes correspond to the outer and inner envelope membranes (oEM and iEM) of the symbiont's chloroplast (**Figure 2**). In plants and photosynthetic cells containing simple plastids, the oEM and iEM have the equipment required for an autonomous synthesis of plastid glycerolipids, and this is possibly the case in diatoms (Boudiere et al., 2012; Boudiere et al., 2014; Petroutsos et al., 2014; Li-Beisson et al., 2019).

Electron microscopy images of cells of nitrogen-starved *P. tricornutum* show tight connections between LDs and the EpM (LD-EpM contact sites), endomembrane vesicles (LD-endomembrane contact sites) and the mitochondria outer envelope membrane (LD-mitochondria contact sites) (Flori et al., 2016) (**Figure 2**). LD-mitochondria contacts are necessary for the consumption of FAs, *via* β-oxidation following TAG hydrolysis, and they are therefore involved in LD degradation rather than biogenesis. Based on proteins detected in the LD of *P. tricornutum* and on the composition of the monolayer of polar glycerolipids limiting this organelle, containing lipids deriving from the plastid, *i.e.* SQDG, and from the ER, *i.e.* PC and DGTA (Lupette et al., 2019), the biogenesis of LDs is likely dependent on a biosynthesis platform at the surface of the EpM or in a endomembrane compartment in the vicinity of the EpM.

The proteome of mature LDs from nitrogen-starved *P. tricornutum* poses questions regarding LD homeostasis. They lack protein markers of LD early formation (such as the *P. tricornutum* SEIPIN orthologue, Phatr3_J47296 (Lu et al., 2017)) or enzymes involved in TAG biosynthesis (such as diacylglycerol acyltransferases, DGATs, or PC:diacylglycerol acyltransferases, PDATs) (Lupette et al., 2019). In numerous eukaryotes including insects, mammals and plants (Bouvier-Nave et al., 2000; Kuerschner et al., 2008; Stone et al., 2009; Wilfling et al., 2013; Ayme et al., 2014) mature LDs were shown to contain the machinery to synthesize TAGs, keeping therefore their ability to expand over time (Yu and Li, 2017). Missing this equipment, mature LDs from diatoms may reach a size limit (**Figure 1**). In organisms such as yeast, connections with the ER remain and proteins from mature LDs can relocate to the ER membrane (Jacquier et al., 2011). We still do not know whether such connections are functional in mature LDs from diatoms and **Figure 1** illustrates the case of a physical separation. Nevertheless, based on LD proteome data, contact sites may exist with the EpM, however these LD-EpM contact sites do not contain TAG synthesizing enzymes (Lupette et al., 2019), which seems contradictory with a role in LD formation. Mature LD-EpM contact sites could possibly operate in a distinct function at late stages, even TAG hydrolysis, as suggested by the presence of a TAG lipase in the EpM, OmTGL, Phatr3_J37711 (Li et al., 2018). Multifunctional LD-membrane contact sites can indeed operate differently, in different metabolic rewiring contexts (Schuldiner and Bohnert, 2017).

LD fusion is also known to occur in multiple eukaryotes, by the action of Cell death-inducing DFF45-like effector (CIDE) family proteins including CIDE-A, CIDE-B, and CIDE-C/Fsp27 (Gao et al., 2017; Yu and Li, 2017; Lv et al., 2019). No CIDE homologue sequence could be identified in the genome of *P. tricornutum* but the presence of structural proteins promoting LD fusion cannot be excluded (**Figure 1**).

In this study we addressed the control of the size of LDs induced by nutrient stress in *P. tricornutum*. We used a pulse field gradient nuclear magnetic resonance (PFG-NMR) method to detect whether mature LDs could be connected by physical bridges allowing LD-to-LD TAG mobility, or ultimately LD-LD fusions. We then analyzed the dynamics of LD size following nitrogen depletion and developed a mathematical model relating LD size limit to constraints dictated by cell geometry and available cytosolic volume.

## Results and Discussion

### Distinct Lipid Droplet Subpopulations Are Generated in *Phaeodactylum tricornutum* Cells Following a Depletion in Nitrogen

To follow the dynamics of LD production, we used nitrogen starvation as a model condition. As reported previously (Abida et al., 2015) a 00N10P ESAW medium, providing ten times the requested amount of phosphate (Pi) needed for *P. tricornutum* growth, is used to define a nitrogen-depleted condition. It ensures that no exhaustion of Pi occurred during the experiment. Consistently with past studies, during 7 days of nitrogen starvation, cell proliferation (growth) of *P. tricornutum* was slower than in nitrogen-rich conditions, the photosynthetic capacity of *P. tricornutum* cells decreased significantly and non-polar lipid accumulation, based on Nile Red staining, increased over time (**Supplemental Figures S1A–C**). Non-photochemical quenching (NPQ) processes help to regulate and protect photosynthesis in environments in which light energy absorption exceeds the capacity for light utilization (Muller et al., 2001). In nitrogen-depleted condition, the NPQ value increased consistently (**Supplemental Figure S1D**).

Glycerolipids from *P. tricornutum* cells were extracted and the total amount of FAs was quantified, showing a regular increase (**Supplemental Figure S2A**). In *P. tricornutum*, main FAs usually contain from 14 carbons and no unsaturations, i.e. 14:0, up to 20 carbon and five double bonds, i.e. 20:5. In the course of the experiment, the proportions of short and medium chain FAs, *i.e.* 14:0, 16:0, and 16:1 increased, whereas the proportion of polyunsaturated and long chain FAs, *i.e.* 16:3, 18:3, and 20:5 decreased (**Supplemental Figure S2B**), reflecting the expected increase in TAG enriched in 16:0 and 16:1.

**Figure 3:**
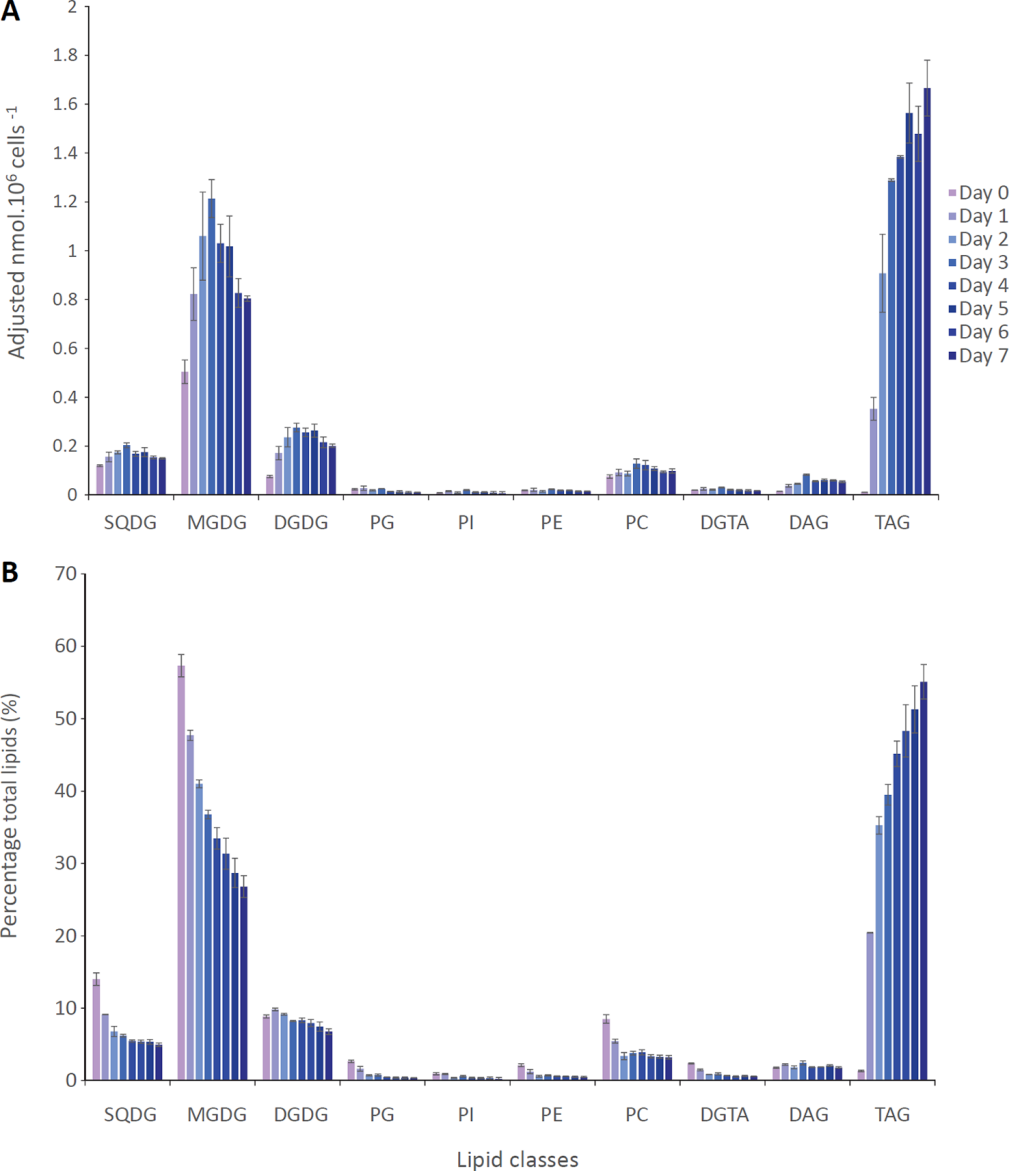
Lipid remodeling in *Phaeodactylum tricornutum* cells grown in nitrogen-depleted condition for 7 days. *P. tricornutum* cells were cultivated in 00N10P ESAW medium as described in the Methods section. **(A)** Quantification of each lipid class in nmol per million of cells. **(B)** Relative proportions of each lipid class in percentage of total glycerolipids. Purple bars correspond to day 0, whereas each day of the time course corresponds to bars from light to dark blue. DAG, diacylglycerol; DGDG, digalactosyldiacylglycerol; DGTA, diacylglyceryl hydroxymethyltrimethyl-β-alanine; MGDG, monogalactosyldiacylglycerol; PC, phosphatidylcholine; PE, phosphatidylethanolamine; PG, phosphatidylglycerol; PI, phosphatidylinositol; SQDG, sulfoquinovosyldiacylglycerol; TAG, triacylglycerol. Data are the average of 3 independent experiments. Error bars show standard deviations.

The content of each glycerolipid class was determined, using liquid chromatography coupled to tandem mass spectrometry (**Figure 3**). In the first 3 days (from day 0 to 3), the amount per million cells of major membrane lipids from photosynthetic membranes, *i.e.* SQDG, MGDG, and DGDG as well as PC, synthesized in the endomembrane system, increase, incorporating FAs, possibly due to an increase in FA synthesis and/or a decrease in their degradation following nitrogen removal (**Supplemental Figure S2A**). In parallel, TAG accumulates (**Figure 3A**). Following this first stage (from day 4 to 7), the quantity per million cells of all these polar lipids decreased, whereas TAG accumulated further, reflecting an arrest of polar lipid synthesis and/or a recycling from polar lipids to TAG (**Figure 3A**). When considering the percentages of the different glycerolipid classes, the proportions of all membrane lipids (with the noticeable exception of DGDG) decreased from the first day of nutrient removal (P-value < 0.05 based on a student's t-test), at the expense of TAG, indicating that the accumulation of TAGs is faster than the transient increase of each membrane lipid (**Figure 3B**). This intense remodeling of membrane and storage glycerolipids is consistent with the functional impairment of the photosynthetic membranes we observed, correlating with the decrease of the photosynthetic capacity, and the accumulation of neutral lipids monitored using Nile Red staining.

**Figure 4:**
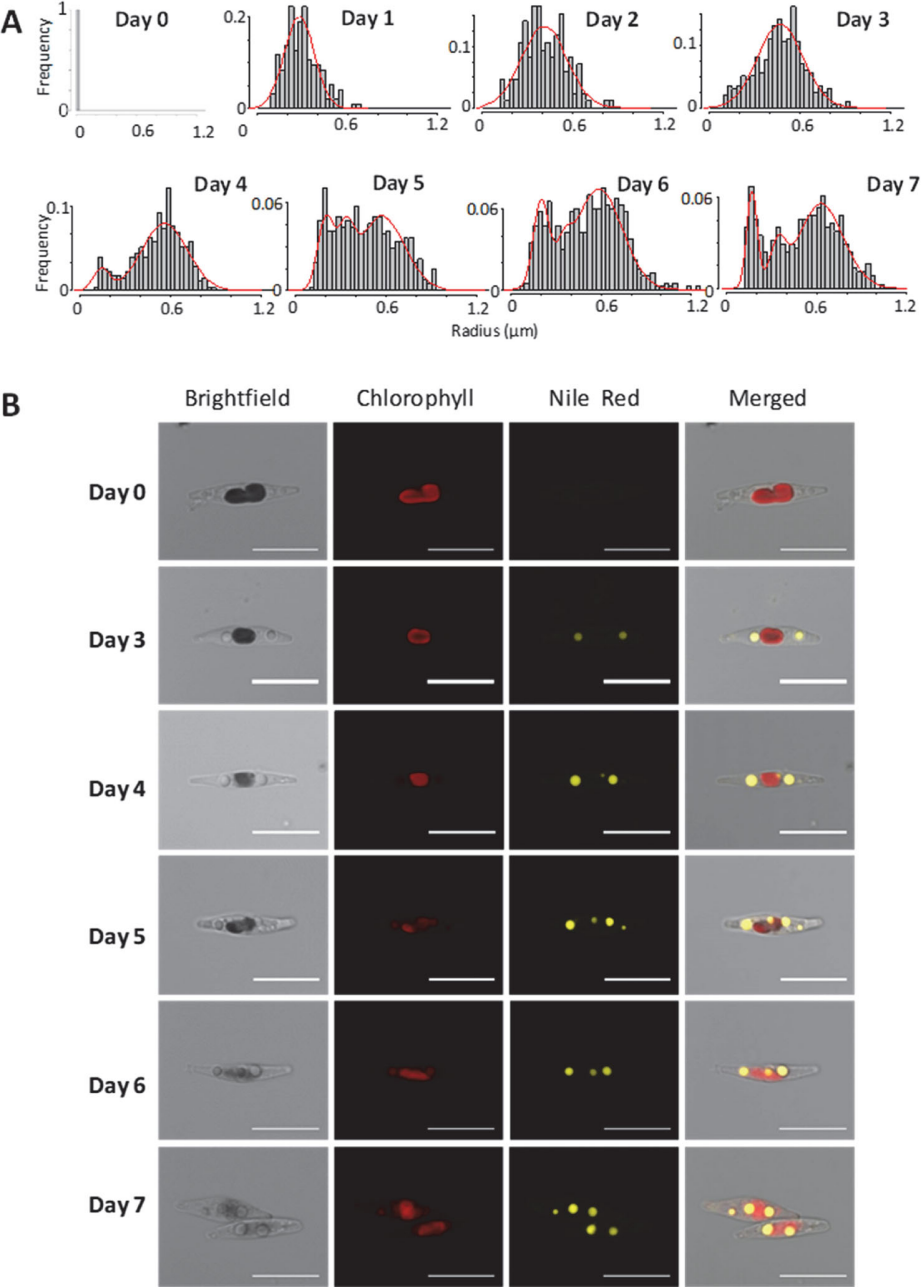
Size of Lipid droplets (LDs) in *Phaeodactylum tricornutum* cells grown in nitrogen-depleted conditions for 7 days. LDs size was determined with an epifluorescence microscope as described in the Methods section. LD measurements were performed on 40, 141, 333, 305, 418, 509, and 373 LDs at day 1 to 7, respectively. The curves shown in red correspond to the predictions based on the mathematical model described at the end of the manuscript. **(A)** Distribution of LD radius measurements. Histograms were based on rounded values to nearest 0.03 µm value. The curve in red shows the predictive model obtained in this work, at the corresponding time value. **(B)** Confocal imaging of representative cells. In red, autofluorescence of chlorophyll (secondary plastid in central position within the cells); in yellow, Nile red staining of LDs.

Based on the staining of LDs by Nile Red, we observed *P. tricornutum* cells by epifluorescence microscopy and measured the size (radius) of LDs in three independent biological triplicates. For each day, there was no statistical difference in size distribution (based on student's t-tests), allowing the analysis of a consolidated distribution of LD sizes measured in 40, 141, 333, 305, 418, 509, and 373 LDs at day 1 to 7, respectively. No cell contained any visible LD at day 0 (**Figure 4A**). Histograms were based on rounded values at the nearest 0.03 µm value. We also visualized cells by confocal microscopy at day 0, and from day 3 to day 6 to detect possible variabilities in cell and LD morphologies (**Figure 4B**).

In the first 3 days, the population of LDs seemed homogenous, with a regular shift of the size (radius value) distribution, from an inexistent radius measurement (day 0) to approximately 0.45 µm (day 1 to 3). At day 4 the overall size distribution shifted to higher radius values, but a second population seemed to appear in lower radius ranges. At day 5, the distribution was broadly dome-like shaped. At day 6, two major populations of radius distributions seemed to appear more distinctly. At day 7, three populations seem to be clearly present with smaller, intermediate and higher radius values (**Figure 4A**). In all samples, the shape of the LDs remained subspherical, with some LDs apparently disconnected (distant) from the secondary plastid, supporting the rupture of direct contact with the EpM, in some of the mature LDs (**Figure 4B**). This analysis of LD size distribution shows therefore that the biogenesis of LDs following a shortage of nitrogen is not a continuous and even process of LD budding, but an ordered production of LD subpopulations, reaching a size limit, with at least three waves during the time of observation, over 7 days.

### Pulse Field Gradient Nuclear Magnetic Resonance Analysis of Triacylglycerol Mobility Highlights the Absence of Lipid Droplet-to-Lipid Droplet Bridges or Lipid Droplet–Lipid Droplet Fusions

**Figure 5:**
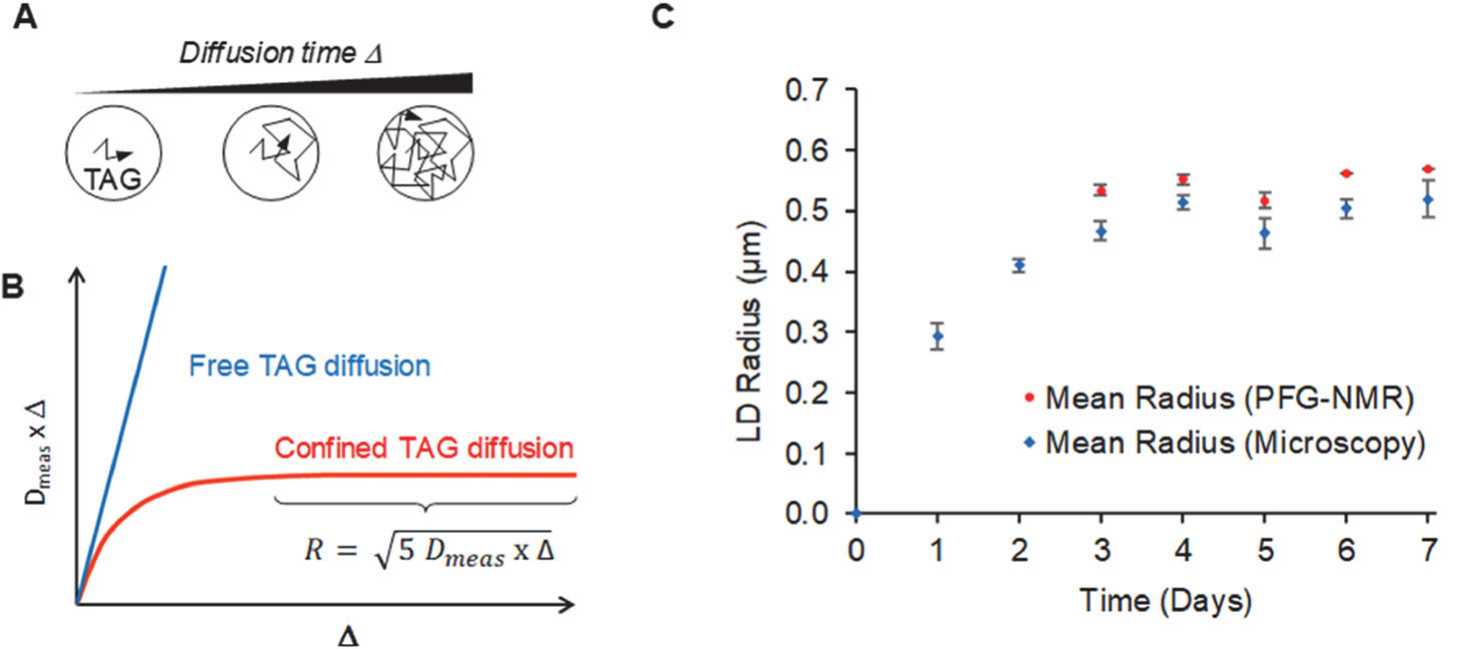
Analysis of TAG confinement inside *Phaeodactylum tricornutum* cells using ^1^H PFG-NMR. **(A)** Schematic definition of *Δ*, the diffusion delay during which a TAG molecular diffusion is observed. Either TAG molecules can diffuse freely, or TAG molecules are confined inside a three dimensional cellular territory, shown here as subspherical. **(B)** Principle of the determination of the size of the cellular territory in which TAG molecules are mobile. When increasing the diffusion delay, either *Dmeas xΔ* is a linear function of *Δ* indicating that TAG is freely mobile without any obstacle, or it reaches a plateau, allowing the determination of the mean radius of the cellular territory inside which TAG is confined. **(C)** Comparison of the mean radius of lipid droplets (LDs) from nitrogen-depleted *Phaeodactylum tricornutum* measured by the analysis of cells by epifluorescence microscopy and by PFG-NMR The measurements of LD radiuses based on PFG-NMR analyses were performed at day 3, 4, 5, 6 and 7 (red points) and compared with values determined with an epifluorescence microscope (blue points). The number of LDs measured in epifluorescence measurements was 40, 141, 333, 305, 418, 509, and 373, for day 1 to 7, respectively. Error bars for Microscopy measurements correspond to standard deviations for mean radiuses calculated in biological triplicates. Error bars for NMR measurements correspond to standard deviations of radius measurements in biological triplicates.

We used a PFG-NMR method previously applied to the determination of TAG mobility inside LDs of seeds (Gromova et al., 2015). This non-invasive approach allows analyzing in a short time the samples containing hundreds of millions of cells. PFG-NMR method is based on measurements of the apparent diffusion coefficient (*D_meas_*) of species (Tanner and Stejskal, 1968), which can differ from the self-diffusion coefficient (*D_self_*) in the case of restricted diffusion. The diffusion delay during which the molecular random motion is analyzed, here that of TAG molecules, is termed *Δ*. For different values of *Δ*, the conditions of free or restricted diffusion can be fulfilled (**Figure 5A**), and different values of *D_meas_* are obtained (*D_meas_ = D_self_* for short *Δ* and *D_meas_ < D_self_* for longer *Δ*). More precisely, these conditions of confined diffusion depend on diffusion time, TAG viscosity and also on the size of confining volume (i.e. LD volume). In the case of confined diffusion, the parameter *D_meas_* x *Δ* corresponding to mean squared displacement of TAG molecules, reaches its limit value (**Figure 5B**), and allows to determine the mean radius of the cell regions in which TAG are confined $\left({\mathrm{Radius}}_{\mathrm{PFG}-\mathrm{NMR}}\;=\sqrt{5D_{\mathrm{meas}}\times \Delta }\right)$ (Tanner and Stejskal, 1968) (**Figure 5B**). As these measurements are based on the attenuation of the spin-echo signal of TAG, and this attenuation is quite small (few percent only) for studied LD size and available field gradient strength, one needs a quite good signal to noise ratio of TAG NMR signal. Consequently, these measurements were performed on algae samples which accumulated a substantial amount of TAG i.e. at the third, fourth, fifth, sixth, and seventh day of the nitrogen starvation. For all samples a plateau values in *D_meas_ xΔ = f(Δ)* plots were observed (**Supplemental Figure 3S**) and the mean Radius values were calculated (**Figure 5C**).

Using PFG-NMR method, we could first determine that TAG molecules are confined inside three-dimensional cellular territories, which themselves do not manifest any displacement at the scale of diffusion time *Δ*. Secondly, the estimated NMR Radius values are very close to those obtained by direct measurements of LDs using epifluorescence microscopy (**Figure 5C**). As expected, NMR Radius values are slightly higher than those obtained by microscopy, because NMR describes a “volume weighted” distribution of LDs (the NMR signal for a given LD being proportional to the number of TAG molecules inside it). The PFG-NMR approach is also expected to be reliable and very robust, because measurements are achieved using millions of cells. It is interesting to note, that the slight decrease in value of the mean radius of LDs observed at day 5 is confirmed here, whereas the two measurements were performed on distinct aliquot fractions. Moreover, the very close values of LD mean size estimated by NMR and by microscopy suggest that the 3D mobility of TAG molecules cannot exceed the volume of one mature LD. Thus, whereas the fusion of mature LDs has been reported in multiple eukaryotic systems (Gao et al., 2017; Yu and Li, 2017; Lv et al., 2019), no evidence could be obtained in *P. tricornutum* that LDs could be connected by bridges allowing LD-to-LD TAG mobility, or ultimately LD-LD fusions. This does not exclude that a portion of LDs may encounter some transient fusion events, but in that case, they would not permit massive movements of TAGs and be, therefore, extremely minor. This result is consistent with the absence of genes coding for homologs of Cell death-inducing DFF45-like effector (CIDE) family proteins (Gao et al., 2017; Yu and Li, 2017; Lv et al., 2019), although the presence of alternative LD fusion systems, operating in other physiological contexts, are not excluded.

### Mathematical Modeling of Lipid Droplet Population Dynamics

Altogether, the analysis of LD size distribution in *P. tricornutum* following a shortage in nitrogen highlights the stepwise generation of subpopulations, at least three, marked by an increase in size (radius) over time reaching a maximum value. These subpopulations are structurally disconnected, without any evidence of LD-LD bridges that would allow the diffusion of TAG from one LD to another. From a structural point of view, *P. tricornutum* cells are very narrow, with a section measuring approximately 15 × 4 µm, containing an ellipsoid plastid of about 6 × 4 µm (Flori et al., 2016; Flori et al., 2018). Taking into account the nucleus and the mitochondria, a very small amount of free-space is left. As a consequence, produced LDs grow until they reach a size limited by cell packing with other membrane organelles and by the stiffness of the limiting cell wall. Considering this observation, and the fact that the mean size of LDs reaches an upper limit at the fourth day (**Figure 5C**), decreasing even slightly on the fifth day, one could assume that a maximum limit might also be reached for TAG accumulation. From the lipidomic profiling results (**Figure 3**), it is clear that the amount of TAG keeps increasing, even after the fourth day, showing inconsistency between Mean radius values for LDs and TAG amount.

The distribution of the LD radius (**Figure 4**) gives insight into a possible explanation for this incoherence. From day 1 to day 3, there is only one population of LDs, which radius increases regularly. From day 4 and after, an additional subpopulation of LD appears, apparently expanding in a more crowded cytosol and reaching therefore a smaller limit value. By multiplying the number of LDs in a same cell, more TAG can be stored, even if the mean LD size does not change.

**Figure 6:**
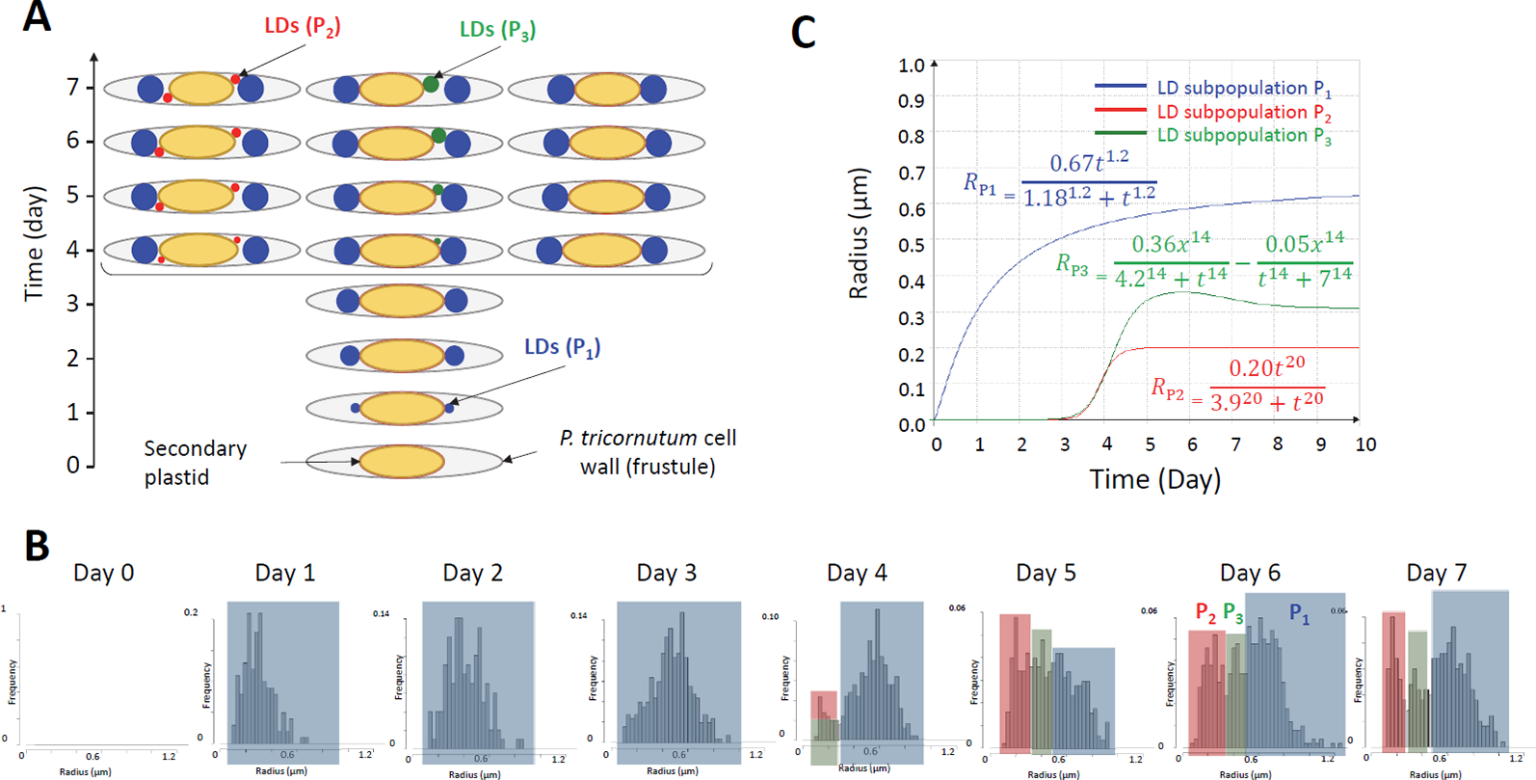
Assumptions for the mathematical simulation of LD dynamics in *Phaeodactylum tricornutum* cells. **(A)** Lipid droplet (LD) subpopulations. The hypothesis is that three distinct subpopulations of LDs are generated over time. P1 (in blue) is the first to appear following transfer of cells into a nitrogen-depleted environment. LDs reach a maximal mean radius at day 3. Then the biogenesis of novel LDs is triggered, generating two subpopulations, P2 (in red) and P3 (in green), which can be distinguished based on the maximal radius they reach, P2 with small LDs and P3 with LDs of intermediate maximal size. Since P3 reaches a higher mean radius than P2, we hypothesize that more cytosolic volume is available to allow LD loading with more TAG. **(B)** Highlighting of subpopulations in LD size distributions. P1, P2 and P3 LD subpopulations are highlighted in blue, red and green respectively. **(C)** Mathematical model. Parameters for the Hill's equations used to simulate the dynamics of P1, P2, and P3 were based on the mean, standard deviation and proportion of each subpopulation.

These observations led to the following hypotheses, as working assumptions for mathematical simulations. In a first hypothesis an initial subpopulation of LDs (P_1_) appears early after nitrogen depletion and the corresponding LD size increases until reaching a maximum at about day 3, then a second subpopulation (P_2_) is produced at day 4, and a third population (P_3_) at day 6. The size of LDs in P_2_ and P_3_ increases until reaching different maxima. Specifically, the radius of LDs of subpopulation P_2_ reaches a maximum at about day 5, and the radius of LDs of subpopulation P_3_ at about day 7. In the second hypothesis (**Figure 6A**), an initial subpopulation of LDs (P_1_) appears early after nitrogen depletion and the corresponding LD size increases until reaching a maximum at day 3, then a second (P_2_) and a third (P_3_) subpopulations are produced at day 4 and the corresponding LDs increase in size until reaching different maxima at different time. Specifically, P_2_ and P_3_ appear at the same time but LDs from subpopulation P_2_ reach a maximum size at about day 3 whereas LDs from subpopulation P_3_ reach their maximum size at day 6. Based on our simulations, only this second hypothesis fitted with real data, and is therefore detailed below.

The main advantage of assuming that distinct subpopulations of LDs are generated is that it explains simply the apparent inconsistency of the increase in the amount of TAG inside cells containing a limited amount of large LDs, having a limited maximal mean radius (subpopulation P_1_). Indeed, additional TAG molecules can be loaded inside smaller LDs corresponding to P_2_ and P_3_ (**Figure 6A, B**). A possible explanation for LDs reaching a maximum radius is the exhaustion of free-space in the cytosol. *P. tricornutum* cells are very narrow organisms bounded by a rigid frustule and containing a secondary plastid, mitochondrion and nucleus which are tightly packed (Flori et al., 2016). Results presented here suggest that once P_1_ has filled available cytosolic space with large size LDs, new LD subpopulations, P_2_ and P_3_ are formed in different locations (**Figure 6A**).

The distribution of P_1_ from day 1 to day 3, and the distributions of the P_2_ and P_3_ subpopulations following day 4, appear to follow a Normal (or Gaussian) distribution (**Figure 6B**). We therefore simulated data evolution using the Hill's equation (Hill, 1910) (**Figure 6C**), with the following equation for a given subpopulation of LDs:

$$R_{LD}=\frac{R_{\max}t^{h}}{t_{1/2}^{h}+t^{h}}$$

With R*_LD_* the radius at time *t*, *R_max_* the size limit, *h* the slope of the curve and *t_1/2_* the time requested to reach *R_max_/2*. Based on this general formula and using observed *R_max_* values for the three subpopulations, P_1_, P_2_ and P_3_ respectively:

$$Mean\;R_{P1}(\mu m)=\frac{0.67t^{1.2}}{1.18^{1.2}+t^{1.2}}$$

$$Mean\;R_{P2}(\mu m)=\frac{0.20t^{20}}{3.9^{20}+t^{20}}$$

$$Mean\;R_{P2}(\mu m)=\frac{0.36x^{14}}{4.2^{14}+t^{14}}-\frac{0.05x^{14}}{t^{14}+7^{14}}$$

The standard deviations are:

$$SD_{P1}=\frac{0,158x^{1,5}}{0,5^{1,5}+\;x^{1,5}}$$

$$SD_{P2}=\frac{0,05x^{23}}{7,15^{23}+\;x^{23}}$$

$$SD_{P3}=0,05-\;\frac{0,05x^{23}}{7,15+\;x^{23}}$$

The simulation of LD size distribution (**Supplemental video 1**) is illustrated at day 6 in **Table 1** and is shown in **Figure 4A**, as a red curve superimposed to the histograms showing the experimental values from day 0 to day 7.

**Table 1:** Comparison of predicted and experimental LD size distribution in *Phaeodactylum tricornutum* cells at day 6, following a depletion of nitrogen in the growth medium.

	Model	Experimental data
Time (Day)	6	6
Mean LD Radius (µm)	0.4942	0.5
TAG (nmol.1 × 10^6^ cells^-1^)	1.43	1.47
LD size distribution	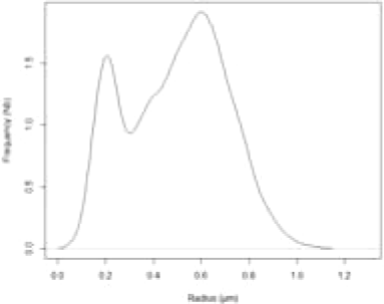	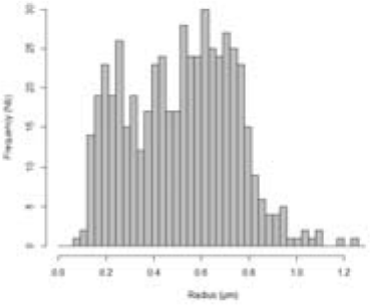

**Figure 7:**
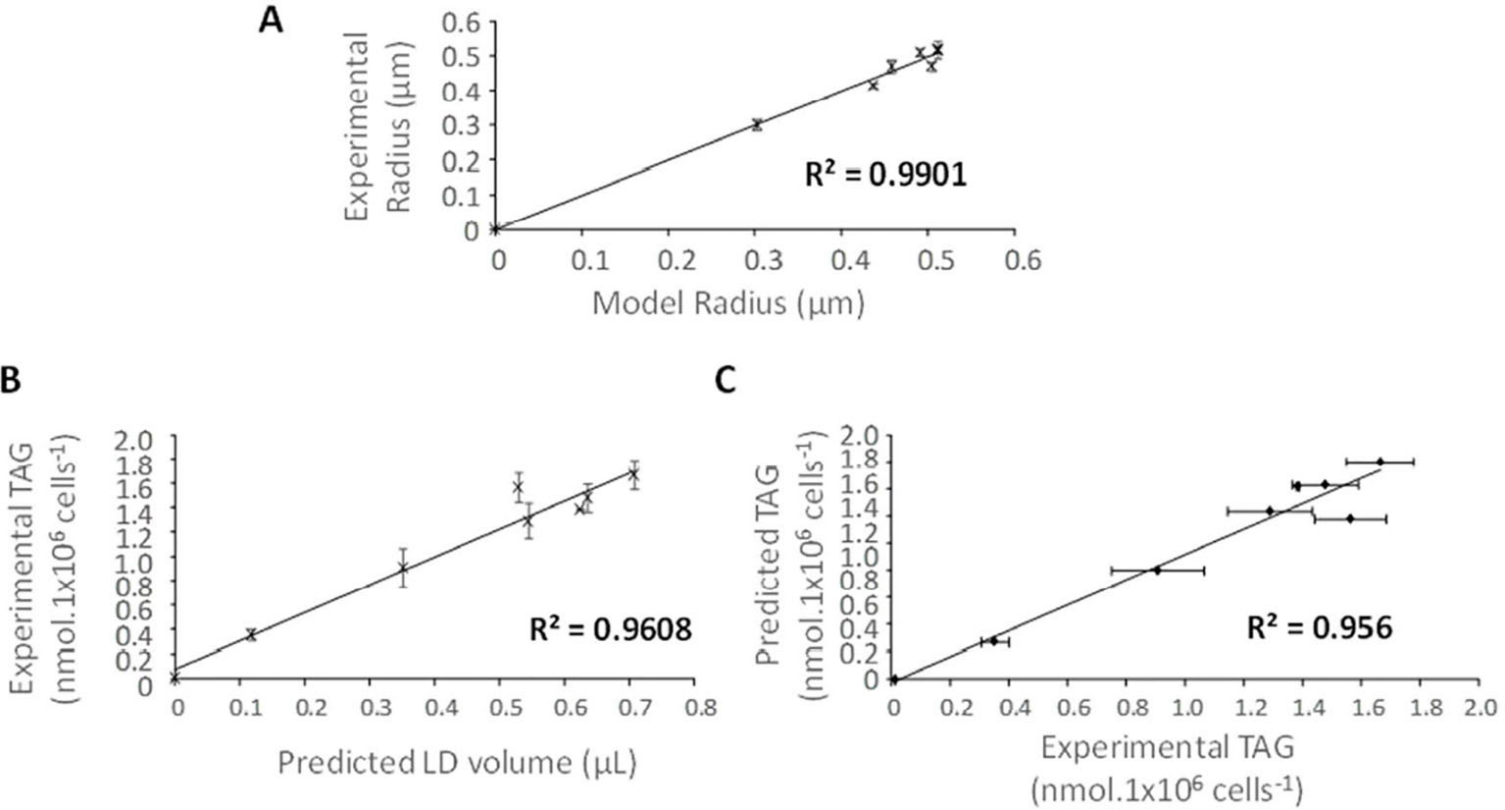
Comparison of the mathematical simulation of LD dynamics in *Phaeodactylum tricornutum* cells with experimental data. **(A)** Correlation between the mean LD radius predicted by the model and the mean LD radius obtained from experimental data. **(B)** Linear correlation between the amount of TAG per million cells and the LD volume predicted by the model. **(C)** Linear correlation between the amounts of TAG determined experimentally and that based on model prediction. Coefficients of determination (R^2^) are indicated based on linear regressions.

When computing mean LD radiuses at day 1 to 7, the model fits linearly to the experimental data with a coefficient of determination R^2^ of 99% (**Figure 7A**). Using the model radius of LDs, it is possible to calculate the corresponding volume: the correlation between the volume predicted by the model and the experimental amount of TAGs is linear, with a coefficient of determination of 96%. Eventually, the correlation between the amount of TAGs predicted by the model based on the correspondence between volume of P_1_ LDs at day 2 with TAG quantification, and the experimental amount of TAGs is also linear, with a coefficient of determination of 95.6% (**Figures 7B, C**).

### Lipid Droplet Subpopulations Have Larger Maximal Sizes in a *P. tricornutum* Mutant Having Larger Cells

**Figure 8:**
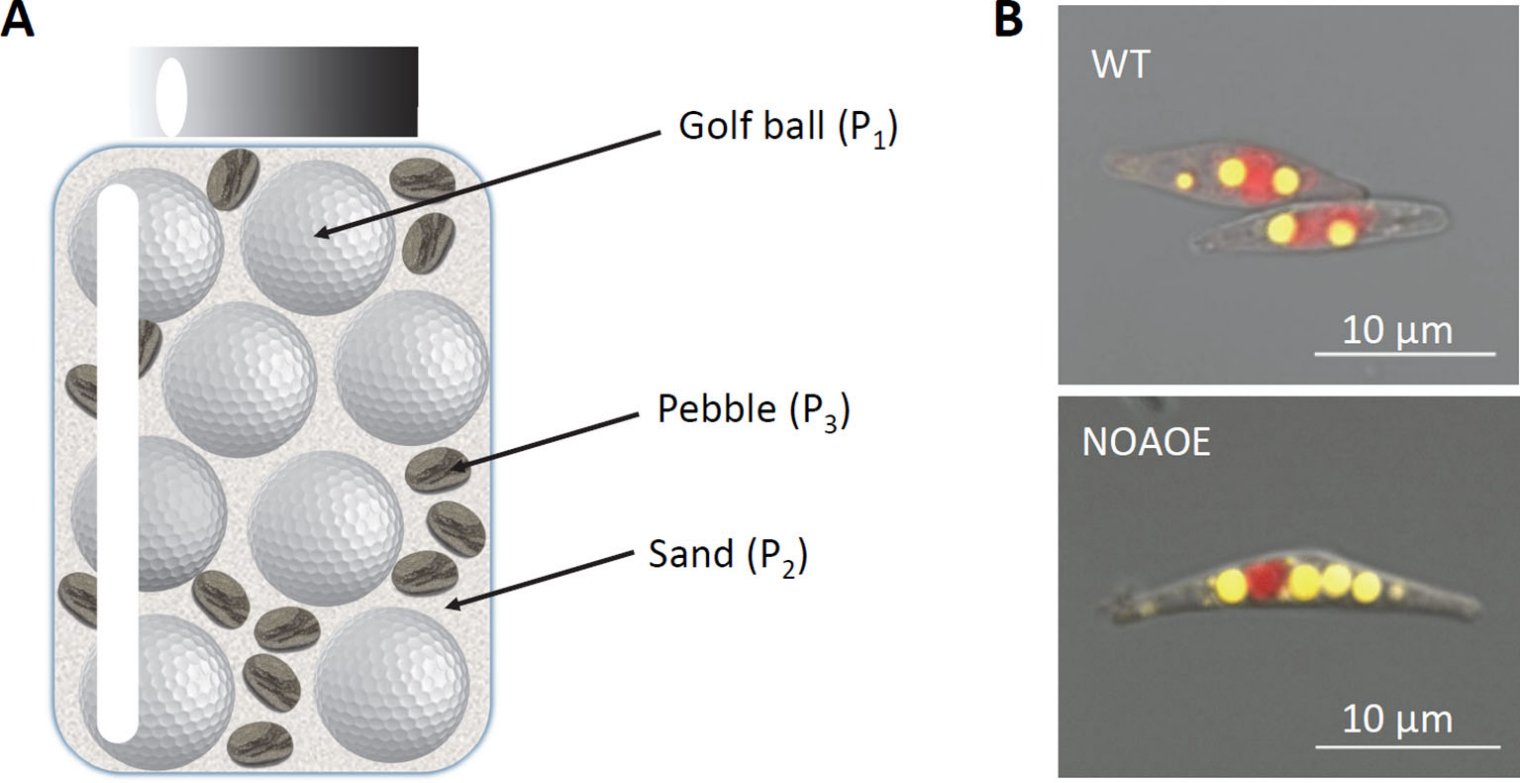
The “jar of life” model. **(A)** Filling of a jar with golf balls, pebbles and sand. In this famous illustration of the way to sort priorities, the most efficient way to fill a jar with golf balls, pebbles and sand is to begin with larger elements, i.e. golf balls, and finish with sand filling the interstices. **(B)** Impact of cell size on LD accumulation? In some mutants of *P. tricornutum*, such as NOA overexpressing lines (NOAOE), cells are larger. These lines have been shown to accumulate more TAG due to a combination of metabolic remodeling and transcriptional reprogramming promoting the synthesis of TAG. The proposed model suggests that their larger cells might contribute to the accumulation of more LDs.

In the ‘jar of life' lesson, a popular metaphor of the way one should handle priorities in a lifetime, a jar is firstly filled with golf balls, then with pebbles and sand (**Figure 8A**). Similarly, LD biogenesis follows a stepwise generation of subpopulations in *P. tricornutum*, reaching large (P_1_), smaller (P_2_), and medium (P_3_) sizes, filling up the available cytosolic space. The radius of P_1_, P_2_ and P_3_ subpopulations reach an average of 0.675 ± 0.125, 0.195 ± 0.046, and 0.385 ± 0.053 µm at day 7, respectively. The lesson of the ‘jar of life' is that if one starts to fill the jar with sand, no more room will be available for pebble and golf balls. Since a sphere has the smallest surface-area-to-volume ratio, the storage of TAGs is the most efficient and less energy demanding in largest LDs (P_1_), leaving cytosolic space for further LDs (P_2_ and P_3_): the stepwise production of subpopulation is therefore the most efficient for diatoms to cope with long stress periods. Based on our observations and model, P_2_ and P_3_ are in fact a unique type of secondary LDs reaching distinct maximal sizes based on the cytosolic space left by P_1_.

Some *P. tricornutum* mutants accumulating more TAG, such as lines overexpressing the NOA gene (NOAOE) (Dolch et al., 2017), have larger cells than wild type (WT) (**Figure 8B**). The expression of NOA was correlated with nitric oxide (NO) emission within the cell, which triggered a transcriptional reprogramming and a metabolic rewiring diverting glycerolipid lipids toward TAG production. We sought whether, based on the present study, NOAOE larger cells could also accommodate LD subpopulations with distinct maximal sizes. The NOAOE mutant line was grown in nitrogen-depleted conditions for 7 days and the size of LDs was determined with an epifluorescence microscope on 327, 211, 194, 415, 438, 529, 508, and 565 LDs at day 0 to 7, respectively.

**Figure 9:**
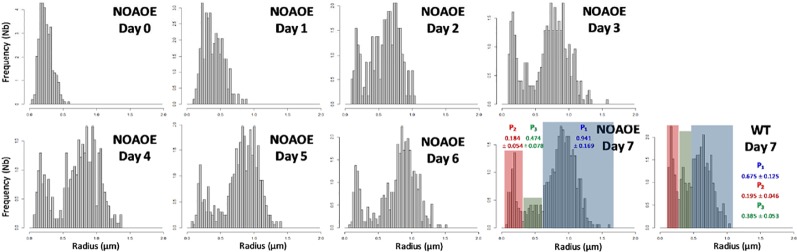
Size of Lipid droplets (LDs) in NOA overexpressing *Phaeodactylum tricornutum* cells (NOAOE) grown in nitrogen-depleted conditions for 7 days. The size of LDs was determined with an epifluorescence microscope as described in the *Material and Methods*. LD measurements were performed on 327, 211, 194, 415, 438, 529, 508, and 565 LDs at day 0 to 7, respectively. Histograms were based on LD radius values rounded to nearest 0.03 µm value. In graphs showing LD distribution at day 7 in WT and mutants cells, the subpopulations are highlighted as in **Figure 6**, with P_1_ in blue, P_2_ in red, and P_3_ in green. The radius mean values for each subpopulation are indicated in the corresponding color. While the mean size of the P_2_ subpopulation is similar between the mutant and WT (p-value = 0.14), there is a significant difference for both P1 and P3 (p-value < 0.001, student's test).

As expected, the NOAOE line contained a basal level of LDs at day 0 (**Figure 9**). Like in WT, three subpopulations were observed, but the P_2_ and P_3_ subpopulations appeared more rapidly, after 2 days rather than 3. The radius of the P_1_ subpopulation measured in NOAOE cells at day 7, with a mean value of 0.941 ± 0.169 µm, was 1.4 times higher than the radius of the P_1_ subpopulation in WT cells (p-value < 0.001). Likewise, the radius of the P3 subpopulation, with a mean value of 0.474 ± 0.078 µm, was 1.2 times higher than that of the P3 subpopulation in WT cells (p-value < 0.001). By contrast, the smaller sized P_2_ subpopulation had a similar size as that in WT, 0.184 ± 0.054 µm. This results confirms that, following the ‘jar of life' model, a first subpopulation of LDs fills up the available cytosolic volume, with a maximal radius determined by the size of the cell. Then smaller sized LDs are produced, filling up available interstices.

## Conclusions and Perspectives

Altogether, this analysis of LD dynamics in *P. tricornutum* following a nitrogen depletion has highlighted key features of LD biogenesis in diatoms, that are consistent with the proteomic and lipidomic architecture recently described (Lupette et al., 2019). Consistently with the lack of TAG synthesizing enzymes in their proteome, such as DGATs or PDATs, mature LDs are not continuously loaded with TAG, do not expand further in size and reach a maximal radius after about 3 days of development. Consistently with the absence of any homologue of CIDE family proteins promoting LD fusion, PFG-NMR analyses of TAG confined diffusion do not show any LD-to-LD bridges or LD-LD fusions that could allow TAG mobility from one LD to another. Our results do not exclude the possibility of LD-LD fusions *via* an alternative mechanism occurring in other conditions.

In WT cells, we observed the stepwise biogenesis of subpopulations of LDs. The P_1_ subpopulation appearing first, consisted of LDs growing in size until reaching a maximal radius. Only after P_1_ LDs reached their size limit, at day 3 in WT cells, two subpopulations appeared, P_2_ and P_3_, of smaller maximal sizes. In a NOAOE mutant line having larger cells, this stepwise biogenesis of LDs was also observed, with NOAOE P_1_ LDs being consistently bigger compared to WT P_1_ LDs. It may be of interest to compare the size of LD subpopulations in other *P. tricornutum* accessions, differing from Pt1 8.6, and to compare fusiform LDs with those generated in triradiate or oval morphotypes. This process may be involved in the formation of LDs with larger sizes in other mutants or physiological contexts. In particular, it has been shown that blocking cell division in diatoms, led to the formation of larger cells, correlating with a higher TAG content (Kim et al., 2017; Conte et al., 2018).

The cellular factor(s) that prevents LD expansion, limiting the size of P_1_, P_2_ and P_3_ at distinct radius magnitudes, seem(s) to be under geometrical and mechanical constraints determined by *P. tricornutum* cellular anatomy, space availability in the cytosol and most importantly cell wall stiffness. It has been shown in animal cells that the Arf1/COPI protein machinery, known for its role in vesicle trafficking, could localize to cytosolic LDs and regulate their morphology and size (Thiam et al., 2013; Wilfling et al., 2014; Olzmann and Carvalho, 2019). In particular Arf1/COPI could lead to the budding of nano-LDs (∼60 nm diameter) from phospholipid-covered oil/water interfaces *in vitro* and were shown to be sufficient to bud nano-LDs from cellular LDs. This process was suggested to be correlated with surface tension in LDs of increasing size and to be associated with a binding of nano-LDs to the ER. Since the Arf1/COPI machinery was also detected in the proteome of *P. tricornutum* LDs (Lupette et al., 2019), the role it may play in the stepwise production of P_2_ and P_3_ subpopulations needs to be investigated. It will also be essential to analyze whether this stepwise biogenetic process operates in response to other stresses, in particular oxidative stresses that were previously shown to trigger the accumulation of smaller LDs containing distinct (more unsaturated) molecular species of TAGs (Lupette et al., 2018). It may be of interest to analyze whether cell space-availability also impacts the dynamics of accumulation of other carbon storage molecules such as carbohydrate polymers, and following which process.

Since oleaginous diatoms have attracted the attention for their potential as a feedstock for applications ranging from food, feed, cosmetics, to biofuels and green chemistry (Levitan et al., 2014; Lupette and Maréchal, 2018; Lupette et al., 2019), expansion of cell size might be considered for biotechnological developments. Future research should therefore focus on the determinant factors of LD-size control, cell shape and cell size in diatoms. It will be essential to evaluate whether this dynamic process of stress LD formation is conserved in other species or whether alternative strategies, including LD-LD fusion processes or mature LD expansion by associated TAG synthesizing enzymes, have been selected in other heterokont clades in the course of evolution.

## Material and Methods

### Cultivation of *Phaeodactylum tricornutum*

*Phaeodactylum tricornutum* (Pt1) Bohlin Strain 8.6 CCMP2561 (Culture Collection of Marine Phytoplankton, now known as NCMA: National Center for Marine Algae and Microbiota) was used in all experiments. Some experiments were also performed with a mutant line overexpressing NOA (Dolch et al., 2017) and referred to as NOAOE. Pt1 and NOAOE cells were maintained and grown in a volume of 1 L in 2-L flasks at 20°C, in a modified ESAW (Enriched Seawater, Artificial Water) medium (NaCl 362.7 mM; Na_2_SO_4_ 25 mM; KCl 8.03 mM; NaHCO_3_ 2.067 mM; KBr 0.725 mM; H_3_BO_3_ 0.372 mM; NaF 0.0657 mM; MgCl_2_ 47.18 mM; CaCl_2_ 9.134 mM; SrCl_2_ 0.082 mM; Na_2_-glycerophosphate 21.8 µM; Na_2_SiO_3_ 105.6 µM; disodium ethylenediaminetetraacetate dehydrate (Na_2_EDTA) 14.86 µM; Fe(NH_4_)_2_(SO_4_)_2_ 5.97 µM; FeCl_3_ 0.592 µM; MnSO_4_ 2.42 µM; ZnSO_4_ 0.254 µM; CoSO_4_ 0.0569 µM; Na_2_MoO_4_ 0.52 µM; H_3_BO_3_ 61.46 µM; Na_2_SeO_3_ 10 nM; biotin (vitamin H) 8.18 nM; cobalamin (vitamin B_12_) 2.94 nM; thiamine (vitamin B_1_) 0.594 µM) (Falciatore et al., 2000) using ten times enriched nitrogen and phosphate sources (10N10P containing 5.49 mM NaNO_3_ and 0.224 mM NaH_3_PO_4_) (Abida et al., 2015). Nitrogen starvation was performed by collecting cells from a 1-L culture by centrifugation at 3,000×*g* for 7 min, and resuspension in 100 ml in the same medium without any addition of NaNO_3_ (00N10P medium). This washing step was repeated three times and cells were then resuspended in 1 L of 00N10P medium, and cultivated for seven additional days. Cells were grown under gentle agitation (100 rpm) on a 12:12 light (40 μE m^-2^_._s^-1^)/dark cycle. Growth was evaluated by cell counting using a TECAN infinite M1000Pro plate reader and determined following the equation *y =* 1.834.10^-08^ *x* + 0.03758, with *x* the absorbance at 730 nm and *y* the number of cells (Conte et al., 2018). All experiments were performed in biological triplicates and monitored using optical microscopy, confocal microscopy and NMR.

### Evaluation of Neutral Lipid Accumulation by Nile Red Staining

Accumulation of TAG droplets was monitored by Nile Red (Sigma Aldrich) fluorescent staining (excitation wavelength at *λ*_ex_ _max__=_ 532 nm and emission at *λ*_em_ _max__=_ 565 nm), as previously described (Cooksey et al., 1987; Abida et al., 2015). In brief, cells were diluted and adjusted to a cell density that was linearly correlated with Nile Red fluorescence. Nile Red solution (40 μl of a 2.5 μg.ml^-1^ stock solution in DMSO) was added to 160 μl cell suspensions, in a well-96 black plate. Fluorescence was measured by spectrophotometry using a TECAN infinite M1000 PRO (*λ*ex = 530 nm).

### Lipid Droplet Size Determination Using Microscopy Acquisition

Three independent biological replicates were subjected to a 7-day nitrogen depletion and analyzed every day to evaluate LD size distributions. For fluorescence microscopy analyses, 6 μl of cell culture stained with Nile Red were used. LDs were visualized using an epifluorescence microscope ZEISS Axio Scope A1 equipped with an EC Plan-NEOFLUAR objective (100x/1.3, oil immersion). Image captures were performed using a Zeiss AxioCam MRc 0.63x camera. Nile Red fluorescence was monitored by excitation at 488 nm and specific emission at 580 nm. Unstained algae cells have a common emission peak at 668 nm, corresponding to the auto fluorescence peak of chlorophyll (red). When stained with Nile Red, yellow fluorescence corresponds therefore to LDs. Acquisitions obtained by epifluorescence microscopy were saved under the Zeiss.czi format and imported with ImageJ 1.48v using the BIO-FORMATS plugins 5.7.2. The scale was predefined in the.czi format, allowing the measurement of LD diameters. Measurements were based on segments drawn from one side to the other of LDs. Data were stored and saved in.txt format and imported with Rstudio 1.1.423v. Diameters were divided by two and transformed into radiuses. Statistical analysis was performed using the computing environment R (https://www.R-project.org), with a significance level of 0.01. Means' comparisons were evaluated using both a Student's test (*n* = number of cells analyzed) and a multiple one-way analysis of variance. The latter was performed with a Bonferonni correction for each day in order to estimate the reliability of the replications. All experimental dataset were tested for normality test on the ANOVA residuals using the Shapiro-Wilk test (Taeger and Kuhnt, 2014). When required, a Bartlett test of homogeneity of variances was also performed. Even with classical data transformations, the LD size in day 4 did not meet ANOVA assumptions and so, the nonparametric counterpart of the one-way analysis of variance, the Kruskall-Wallis test, was applied. For confocal microscopy imaging, 3 µL of stained *P. tricornutum* suspensions were used. Observations were carried out using a Zeiss LSM800 confocal laser scanning microscope equipped with a Zeiss Plan-APO objective (x63/1.46, oil immersion) and enlarged four times. Nile Red fluorescence was monitored by excitation at 488 nm and capture zone ranging from 579 to 641 nm. Chlorophyll fluorescence was monitored by excitation at 488 nm and capture zone ranging from 650 to 700 nm. Bright field acquisitions are also performed.

### Photosynthetic Capacity (F_v_/F_m_) and Non-Photochemical Quenching

The Fv/Fm ratio was used as an indicator of Photosytem II activity in a dark-adapted state. *In vivo* chlorophyll fluorescence was determined using a Speedzen MX fluorescence imaging system (JBeamBio) with settings previously described (Johnson et al., 2009; Allorent et al., 2013). To this end, a 140 µl volume of *P. tricornutum* culture was transferred to a transparent 96-well plate and dark-incubated for 15–30 min before measurements. Excitation was performed in the blue range (*λ* = 450 nm, *F_0_*). *F_0_* corresponds to the steady state fluorescence in dark-adapted cultures, *F_m_* to the maximal fluorescence after a saturating light pulse with green light (520 nm) of dark-adapted cultures, *F_m_*' the same in light adapted cultures, and *F_v_* the difference between *F_m_* and *F*0. With these parameters, the maximum efficiency of energy conversion of photosystem II (PSII) can be calculated as *F_v_/F_m_* (Butler and Kitajima, 1975; Genty et al., 1990; Misra et al., 2012)V. A sequence of saturating flashes was applied on top of the actinic light to probe NPQ (Muller et al., 2001).

### Glycerolipid Analyses

Whole cell lipids were extracted using the Folch method (Folch et al., 1957; Simionato et al., 2013). In brief, freeze-dried cells were suspended in 4 ml of boiling ethanol for 5 min to prevent lipid degradation, and lipids were extracted by addition of 2 ml methanol and 8 ml chloroform at room temperature. The mixture was then saturated with argon and stirred for 1 h at room temperature. After filtration through glass wool, cell debris were rinsed with 3 ml chloroform/methanol 2:1, v/v, and 5 ml of NaCl 1% were added to the filtrate to initiate phase separation. The chloroform phase was dried under argon before solubilizing the lipid extract in 1 mL of chloroform. Extracted lipids were dried under a flow of argon and conserved at -20°C until analyses. For each lipid extract, total glycerolipids were quantified from their FAs: in a 10 µl aliquot fraction a known quantity of saturated 15-carbon FA (15:0) was added and all FAs were methanolyzed into methyl esters (FAME) by a 1 hour incubation in 3 ml 2.5% H_2_SO_4_ in pure methanol at 100°C (Jouhet et al., 2003). The reaction was stopped by addition of 3 ml water, and 3 ml hexane was added for phase separation. After 20 min of incubation, the hexane phase was transferred to a new tube. FAMEs were extracted a second time *via* the addition, incubation and extraction of another 3 ml hexane. The combined collected hexane fractions (6 ml) were argon-dried and FAMEs were suspended in 40 µl hexane for analysis by gas chromatography coupled with flame ionization detection (GC-FID) (Perkin Elmer), using a BPX70 (SGE) column. FAMEs were identified by comparison of their retention times with those of standards (Sigma) and quantified by the surface peak method using 15:0 for calibration. Extraction and quantification were performed with three biological replicates. Glycerolipids were then analyzed and quantified by high-pressure liquid chromatography-tandem mass spectrometry (HPLC-MS/MS), with appropriate standard lipids (Jouhet et al., 2017). In brief, lipid extracts corresponding to 25 nmol of total FAs were dissolved in 100 µl of chloroform/methanol [2/1, v/v] containing 125 pmol of each internal standard. Internal standards used were phosphatidylethanolamine (PE) 18:0–18:0 and diacylglycerol (DAG) 18:0–22:6 from Avanti Polar Lipid, and SQDG 16:0-18:0 extracted from spinach thylakoid (Deme et al., 2014) and hydrogenated (Buseman et al., 2006). Lipids were then separated by HPLC and quantified by MS/MS. Lipid classes were separated using an Agilent 1200 HPLC system using a 150 × 3 mm (length × internal diameter) 5 µm diol column (Macherey-Nagel), at 40°C. The mobile phases consisted of hexane/isopropanol/water/1 M ammonium acetate, pH 5.3 [625/350/24/1, v/v] (A) and isopropanol/water/1 M ammonium acetate, pH 5.3 [850/149/1, v/v] (B). The injection volume was 20 µL. After 5 min, the percentage of B was increased linearly from 0 to 100% in 30 min and kept at 100% for 15 min. This elution sequence was followed by a return to 100% A in 5 min and an equilibration for 20 min with 100% A before the next injection, leading to a total runtime of 70 min. The flow rate of the mobile phase was 200 µl/min. The distinct glycerophospholipid classes were eluted successively as a function of the polar head group. Mass spectrometric analysis was performed on a 6460 triple quadrupole mass spectrometer (Agilent) equipped with a Jet stream electrospray ion source under following settings: drying gas heater at 260°C, drying gas flow at 13 L.min^-1^, sheath gas heater at 300°C, sheath gas flow at 11 L.min^-1^, nebulizer pressure at 25 psi, capillary voltage at ±5,000 V and nozzle voltage at ±1,000 V. Nitrogen was used as collision gas. The quadrupoles Q1 and Q3 were operated at widest and unit resolution respectively. PC and DGTA analyses were carried out in positive ion mode by scanning for precursors of m/z 184 and 236 respectively at a collision energy (CE) of 34 and 52 eV. SQDG analysis was carried out in negative ion mode by scanning for precursors of m/z -225 at a CE of -56eV. Phosphatidylethanolamine (PE), phosphatidylinositol (PI), phosphatidylglycerol (PG), MGDG, and DGDG measurements were performed in positive ion mode by scanning for neutral losses of 141, 277, 189, 179, and 341 Da at CEs of 20, 12, 16, 8, and 8 eV, respectively. DAG and TAG species were identified and quantified by multiple reaction monitoring (MRM) as singly charged ions [M+NH4]+ at a CE of 16 and 22 eV respectively. Quantification was done for each lipid species by multiple reaction monitoring (MRM) with 50 ms dwell time with the various transitions previously recorded (Abida et al., 2015). Mass spectra were processed using the MassHunter Workstation software (Agilent) for identification and quantification of lipids. Lipid amounts (pmol) were corrected for response differences between internal standards and endogenous lipids and by comparison with a qualified control (QC). QC extract correspond to a known *P. tricornutum* lipid extract qualified and quantified by thin-layer chromatography and gas-chromatography coupled to ion flame detection as described previously (Abida et al., 2015).

### Pulse Field Gradient Nuclear Magnetic Resonance

For NMR analyses of *P. tricornutum* LDs, three independent biological replicates were subjected to a 7-day nitrogen depletion. From day 3 to day 7, 100-ml cell culture samples were centrifuged at a maximum speed of 3,500×*g* and obtained pellets of about 200 µl were transferred gently into 5 mm Shigemi NMR tubes. The high cell concentration ensures the immobility of cells during NMR experiments. All PFG-NMR analyses were performed at 4°C with a Bruker AVANCE spectrometer operating at 500.18 MHz for ^1^H, and equipped with a 5 mm BBI-xyz-gradient probe. Measurements of average LD size were performed by PFG-NMR method as described previously (Gromova et al., 2015). The ^1^H diffusion filtered spectra were recorded using the standard stimulated echo pulse sequence with bipolar gradient (Wu et al., 1995). The diffusion coefficients of observed species were determined according to the Stejskal−Tanner equation:

$$\frac{\text{I}}{{\text{I}}_{0}}={\text{e}}^{-{\left({\text{γ}}_{H}\text{gδ}\right)}^{2}\left(\text{Δ }-\frac{\text{δ}}{3}-\frac{\text{τ}}{4}\right)\times \text{D}}$$

Applied to LD size measurement, *I* and *I_0_* were the integrals of TAG NMR signals with and without applied gradient, respectively. The parameter *γ* is the ^1^H gyromagnetic ratio. The main parameters of the experiment are on the one hand the strength (*g*) and the duration (*δ*) of field gradient pulses and on the other hand the diffusion delay *Δ* during which the molecular diffusion is observed. The delay *τ* is a short time interval between the two pulses in the bipolar gradient modules of the sequence. The parameters of NMR experiments were chosen in order to ensure the conditions of confined diffusion of TAG and the disappearance of water and cytoplasmic metabolites signals due to their higher diffusivity. The amplitude of the trapezoidal gradient pulses (g) was varied from 30 to 98% of the maximum amplitude of 0.48 T.m^-1^, the rise time and fall time of gradient pulses were equal to 10% of the total pulse duration. The experiments were carried out with *δ* = 5 ms, *τ =* 0.2 ms and *Δ* varying from 150 to 450 ms. At those values of *Δ* the conditions for confined diffusion of TAG were reached; consequently, the measurement of diffusion coefficient of TAG molecules (*D_meas_*) allowed to determine the mean radius of cell regions or territories in which TAG were confined $\left(Radius=\sqrt{5D_{meas}\times \Delta }\right)$ (Tanner and Stejskal, 1968). For a given distribution of LD sizes, the measured radius rather represent the mean radius of volume-weighted distribution of spherical LD (Guillermo and Bardet, 2007). In this work, typically, 8 or 16 acquisition scans were performed for each of the 10 gradient values used to measure the echo attenuation and calculate *D_meas_.* For each algae sample, the *D_meas_* were determined for 5 to 7 different values of $\tilde{\Delta }$ The amplitude of the PFG-NMR signal was measured by integrating a part of TAG (-CH_2_-)_n_ protons signal between 1.4 and 1.1 ppm. The observed NMR signals of TAG in algae are widely broadened due to the heterogeneity of the sample. Nevertheless, this peak broadening does not prevent the measurement of TAG signal attenuation, since the TAG signal represent almost the only detected signal in algae diffusion filtered spectra recorded with appropriate choice of parameters (**Supplemental Figures S3** and **S4**).

## Data Availability Statement

All datasets generated for this study are included in the article/**Supplementary Material**.

## Author Contributions

AJ and JL performed most of the experiments and contributed equally to this work. JS provided expertise in the analysis of mutant lines. JJ provided specific expertise in glycerolipid analyses. OB provided expertise in mathematical modelling and statistics. MG and EM conceived the project. All the authors contributed to the writing of the article.

## Funding

This work was supported by a DRF Impulsion program from CEA (RMN-Biocar), a Flagship program from the CEA High Commissioner and the French National Research Agency (Oceanomics ANR-11-BTBR-0008, GlycoAlps ANR-15-IDEX-02, GRAL Labex ANR-10-LABEX-04, and EUR CBS ANR-17-EURE-0003).

## Conflict of Interest

The authors declare that the research was conducted in the absence of any commercial or financial relationships that could be construed as a potential conflict of interest.

## Supplementary Material

The Supplementary Material for this article can be found online at: https://www.frontiersin.org/articles/10.3389/fpls.2020.00048/full#supplementary-material
