# Supplementary material for: Stepwise Biogenesis of Subpopulations of Lipid Droplets in Nitrogen Starved Phaeodactylum tricornutum Cells
Source: Front Plant Sci. 2020 Feb 11;11:48. doi: 10.3389/fpls.2020.00048 (PMC7026457; doi:10.3389/fpls.2020.00048)
Supplement: Supplementary file 1 [file DataSheet_1.pdf]

Stepwise biogenesis of subpopulations of lipid droplets in nitrogen starved *Phaeodactylum tricornutum* cells

Antoine Jaussaud et al.

Supplementary Figures

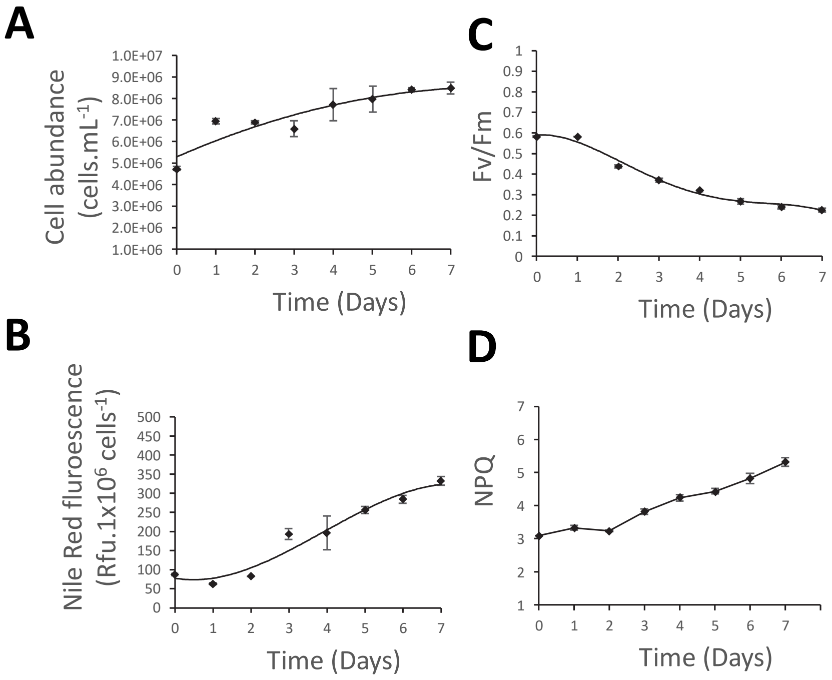

Supplementary Figure S1. Physiological analysis of *Phaeodactylum tricornutum* grown in a shortage of nitrogen (00N10P ESAW medium) for 7 days. A, Growth curve of *P. tricornutum*. B, Time-course analysis of the accumulation of non-polar lipids based on Nile Red staining, expressed in relative fluorescence unit (Rfu). C, Evolution of photosynthetic capacity (Photosystem II). The Fv/Fm ratio was used as an indicator of Photosytem II activity in a dark-adapted state. Excitation was performed in the blue range (λ = 450 nm, F0). F0 corresponds to the steady state fluorescence in dark-adapted cultures, Fm to the maximal fluorescence after a saturating light pulse with green light (520 nm) of dark-adapted cultures, Fm' the same in light adapted cultures, and Fv the difference between Fm and F0. With these parameters, the maximum efficiency of energy conversion of photosystem II (PSII) can be calculated as Fv/Fm (see Methods). D, Non-Photochemical Quenching (NPQ) measurements.

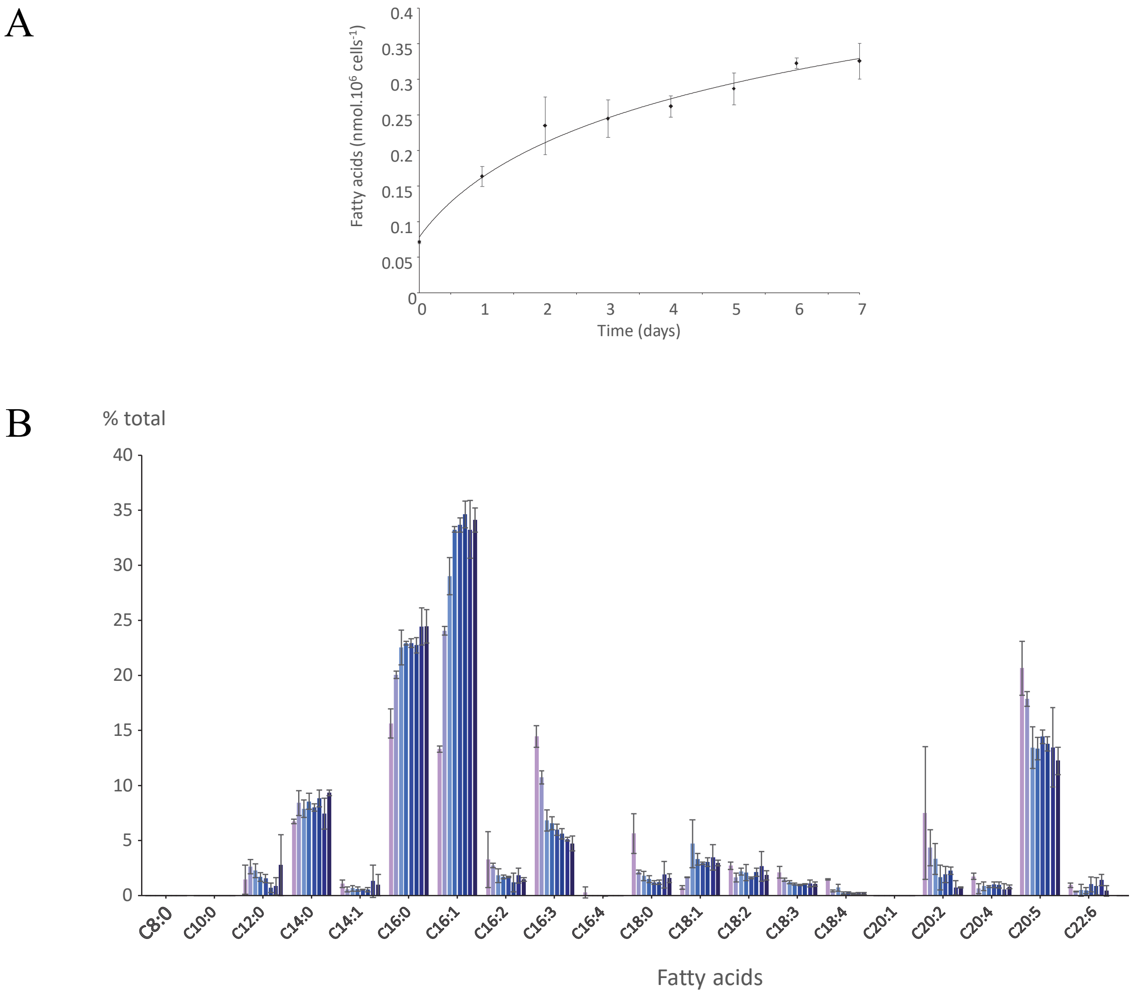

Supplementary Figure S2. Quantification and analysis of total fatty acids extracted from *Phaeodactylum tricornutum* cells grown in nitrogen-depleted conditions (00N10P ESAW) for seven days. A, Fatty acids contents were determined as described in the Methods section, after methanolysis into fatty acid methyl esters (FAMEs) and quantification using gas chromatography coupled to flame ionization detector (GC-FID). B, Fatty Acid profiles were determined based on GC-FID chromatograms. Purple bars correspond to Day 0, whereas each day of the time course corresponds to bars from light to dark blue.

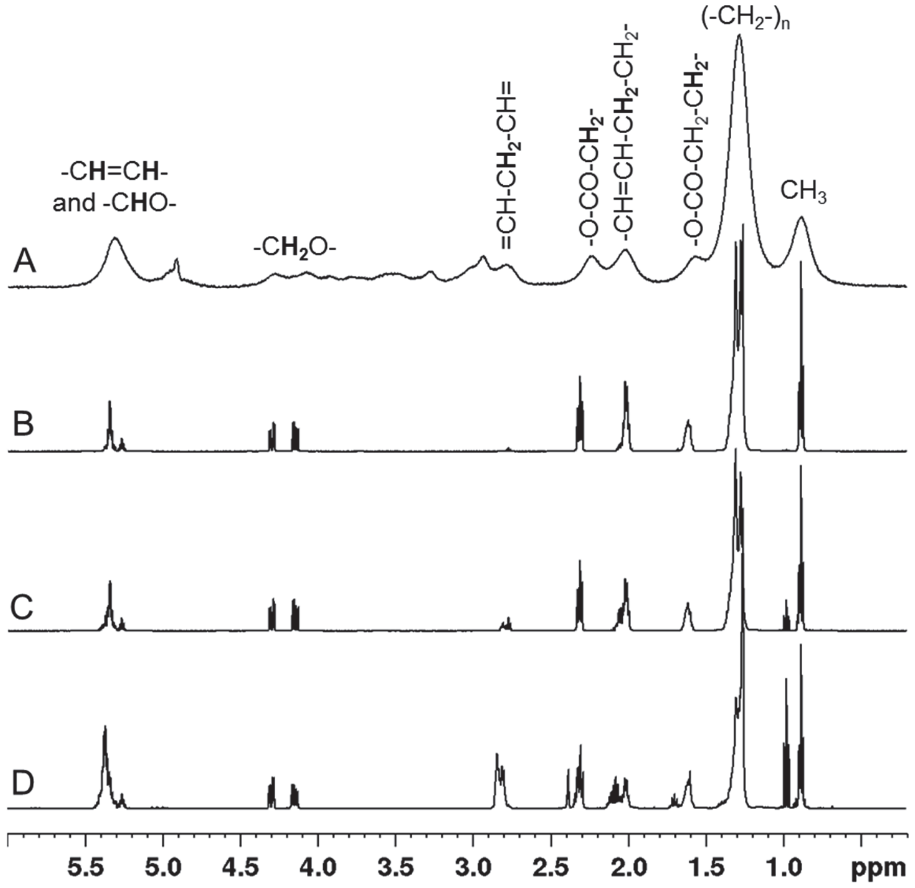

Supplementary Figure S3: ^{1}H NMR spectra recorded at 500 MHz. A) Diffusion filtered NMR spectrum of 7 days N-limited algae cells (Δ= 400 ms, δ=5ms, g= 98% of the maximum value of 0.48 $T.m^{-1}$, number of scans=32). B) - D) NMR spectra of oil in $CDCl_3$: olive oil (B), colza oil (C), fish oil (D)

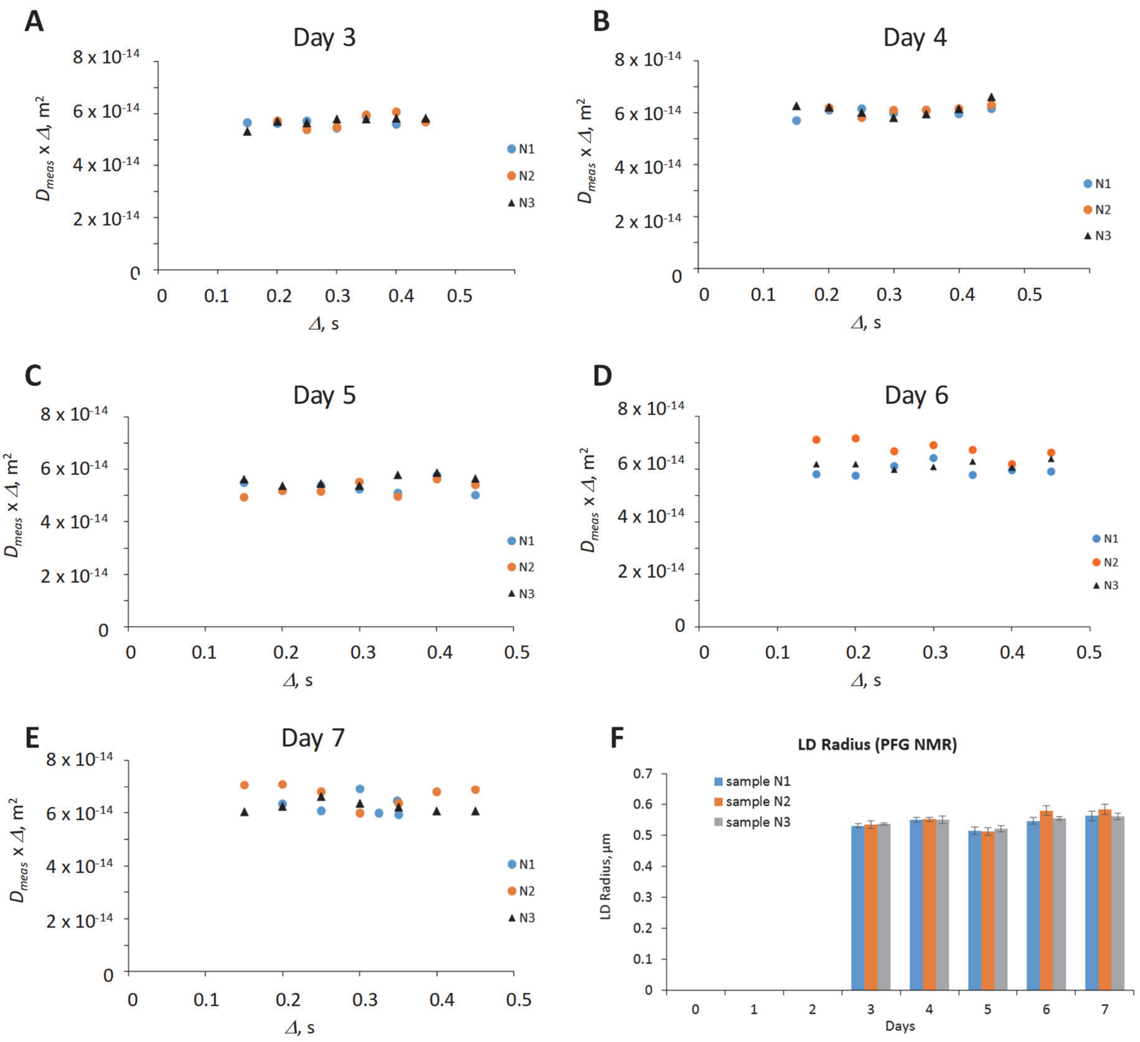

Supplementary Figure S4: (A) to (E) PFG-NMR LD size determination for 3 to 7 days of nitrogen starvation (biological triplicate N1, N2 and N3). The observed plateau for $D_{meas}\ x\ \Delta = f(\Delta)$ plots (see Figure 5B in main text) proves that TAG are confined inside unconnected lipid droplets. (D) Calculated values of mean LD Radius ($Radius\ = \sqrt{5D_{meas} \times \Delta}$) in each replicate.
